# Amino acid intake with protein food source and incident dyslipidemia in Korean adults from the Ansan and Ansung Study and the Health Examinee Study

**DOI:** 10.3389/fnut.2023.1195349

**Published:** 2023-07-21

**Authors:** Sangwon Chung, Jae Ho Park, Hyojee Joung, Kyungho Ha, Sangah Shin

**Affiliations:** ^1^Personalized Diet Research Group, Korea Food Research Institute, Wanju-gun, Jeollabuk-do, Republic of Korea; ^2^Department of Public Health, Graduate School of Public Health, Seoul National University, Seoul, Republic of Korea; ^3^Department of Food Science and Nutrition, Jeju National University, Jeju, Republic of Korea; ^4^Department of Food and Nutrition, Chung-Ang University, Ansung-si, Gyeonggi-do, Republic of Korea

**Keywords:** amino acid, plant-based protein, animal-based protein, dyslipidemia, cohort, Korea

## Abstract

**Background:**

Dyslipidemia is a major risk factor for cardiovascular diseases and appropriate intake of amino acids may be helpful for the management of dyslipidemia. However, evidence of an association between amino acid intake and dyslipidemia in Korean adults is limited.

**Objective:**

The purpose of this study was to investigate how the incidence of dyslipidemia in Korean adults is associated with the consumption of amino acids, essential and nonessential types, as well as the sources of these amino acids from food.

**Methods:**

Data from 35,478 study participants without dyslipidemia at baseline from the Ansan and Ansung Study and the Health Examinee Study were used for the analysis. Dyslipidemia and its components such as hypertriglyceridemia, hypercholesterolemia, hyper-low-density lipoprotein (LDL) cholesterolemia and hypo-high-density lipoprotein (HDL) cholesterolemia were the main outcome in this study. The participants were categorized into quartiles, based on the intake of amino acids and plant−/animal-based proteins.

**Results:**

On average, the follow-up period lasted for 5.7 years. The two major food groups that contributed to one-half of the intake for each type of amino acid were whole grain mixed rice and white rice. Compared to the lowest quartile group, the highest quartile groups of essential amino acid intake [men: hazard ratio (HR) = 0.78; 95% confidence interval (CI), 0.63–0.97; *P* for trend = 0.0088; women: HR = 0.86; 95% CI, 0.76–0.99; *P* for trend = 0.0201] and nonessential amino acid intake (men: HR = 0.75; 95% CI, 0.60–0.94; *P* for trend = 0.0069; women: HR = 0.81; 95% CI, 0.71–0.93; *P* for trend = 0.0024) had a decreased risk of dyslipidemia. Plant-based protein intake had a negative association and animal-based protein intake had a nonsignificant association with dyslipidemia after adjustment for energy-adjusted fat intake. Furthermore, the essential and nonessential amino acid intake showed stronger negative associations with dyslipidemia after further adjustment for energy-adjusted fat intake.

**Conclusion:**

To conclude, the intake of amino acids may have a protective effect against dyslipidemia in Korean adults who are aged 40 years or older, regardless of their protein food sources.

## 1. Introduction

Dyslipidemia, which can lead to stroke, coronary heart disease, and ischemic heart disease, is a significant risk factor for cardiovascular diseases (CVDs) and can influence their incidence (1). Increased cholesterol levels in 20- to 39-year-old young adults have been associated with a high risk of ischemic heart disease incidence (2). In addition, more than 40 and 20% of global death by ischemic heart disease and stroke, respectively, were attributed to elevated levels of plasma low-density lipoprotein cholesterol (LDL-C) (3). However, the improvement of dyslipidemia, such as lowered triglyceride (TG) levels, has been associated with decreased CVD risk in previous studies (4). Therefore, paying close attention to the management and prevention of dyslipidemia as a means to enhance health and increase longevity is crucial.

Healthy dietary habits are recommended as a strategy for lowering lipid levels and preventing CVD (5, 6). Maintaining a balanced intake of energy sources, including an appropriate proportion of protein in one’s diet, has been a long-standing concern for managing various diseases and is crucial for a healthy diet (7). Although the results remain inconclusive, several studies have shown that a high protein intake improves cardiometabolic parameters, including lipid profiles, blood pressure, and glycemic regulation (8–10). Moreover, an appropriate intake of amino acids can be helpful for the management of cardiometabolic health has been reported.

Amino acids are components of proteins and are traditionally categorized as essential and nonessential amino acids. Consuming essential amino acids from dietary sources is essential because the carbon skeletons of these amino acids cannot be synthesized *de novo* or they are inadequately synthesized by cells to meet the metabolic needs of the body (11). In general, animal-based foods have sufficient essential amino acids, and several plant-based foods have a limited quantity of essential amino acids (12, 13). Thus, having sufficient essential amino acids from various food sources is important, especially in the Korean diet, which is primarily composed of plant-based foods.

Each amino acid has different metabolic functions (14, 15). Studies (16–18) have confirmed that the intake of several essential amino acids such as arginine, glutamine, and histidine, and the nonessential amino acids such as cysteine and glycine improve endothelial dysfunction and vascular stiffness and reduces blood pressure. In another study (19), the levels of plasma free essential amino acids including leucine, isoleucine, valine, tyrosine, and phenylalanine were positively associated with metabolic diseases, including dyslipidemia, in a large Asian population study. Investigators have also reported that the physiological effects of amino acids from different protein food sources can differentially affect cardiovascular health (15, 20). Nevertheless, limited evidence exists regarding the impact of amino acid intake, based on their type and food sources and their functions, on dyslipidemia in a large population.

The association between amino acid intake and health has been explored in the elderly Korean population (21–23). In several cross-sectional studies (21, 22), branched-chain amino acid intake was associated with high skeletal muscle mass in older adults aged 50–64 years. However, the amino acid intake of elderly individuals may differ from that of young and middle-aged adults. Based on the findings of previous studies, individuals aged 60 years or older consumed only 30% of their protein from animal-based foods (23, 24), whereas animal-based protein intake among adults aged 30–64 has increased (25). Moreover, the association of other types of amino acid intake and food sources with dyslipidemia in younger adult populations has rarely been estimated. As a result, our objective was to investigate the association between dietary intake of amino acid, based on the amino acid type and food sources, and the occurrence of dyslipidemia in Korean adults who are aged 40 years or older.

## 2. Methods

### 2.1. Study population

**Figure 1:**
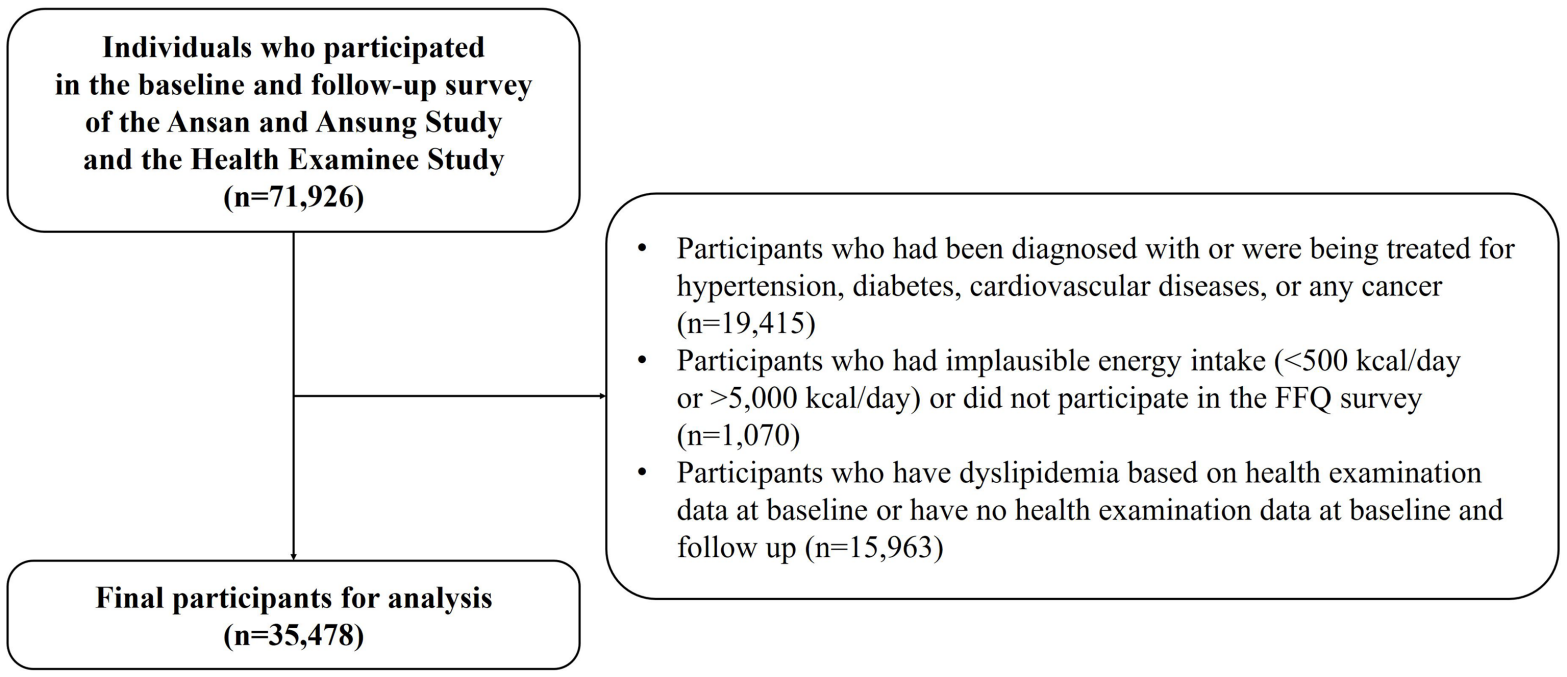
Flow chart of the participants used in this study from the Ansan and Ansung Study and the Health Examinee Study in Korea.

We used data from the Ansan and Ansung Study and the Health Examinee (HEXA) Study in Korea. In brief, these studies are part of the Korean Genome and Epidemiology Study (KoGES), conducted by the Korea Disease Control and Prevention Agency (Cheongju, Republic of Korea) with the aim of exploring the effects of diet, lifestyle, and environmental factors on chronic diseases in the Korean population. The Ansan and Ansung Study recruited 10,030 participants, aged 40–69 years, from urban and rural regions, Ansan and Ansung, respectively, in Korea. Data were examined from 2001 to 2002. The HEXA study recruited 173,195 participants, aged ≥40 years, from 38 health examination centers or hospitals in urban areas across the country. Baseline data were obtained from 2004 to 2013. Comprehensive information on the cohorts is available elsewhere (26). We used data from the baseline to the follow-up in 2012–2017. Among 71,926 individuals who participated in the baseline and follow-up survey, individuals were excluded who were diagnosed with or were being treated for hypertension, diabetes, CVDs, or any cancer (n = 19,415); had implausible energy intake (<500 kcal/day or > 5,000 kcal/day) or no information on the Food Frequency Questionnaire (FFQ) survey (n = 1,070); or had dyslipidemia, based on health examination data at baseline or had no health examination data at baseline and follow-up (*n* = 15,963; Figure 1). Finally, the data of 35,478 study participants (10,012 men and 25,466 women) were used for the analysis. The institutional review board of Chung-Ang University (Ansung, Republic of Korea; approval no., 1041078-202109-HR-278-01) approved this study protocol. Written informed consent was provided by each participant.

### 2.2. Dietary data assessment

Data from the validated semi-quantitative FFQ (27) were used to estimate amino acids, protein, and food intake. The amino acid database was expanded, using the Korean Food Composition Table of the Rural Development Administration (28), the Food and Nutrition Composition Database of the Ministry of Food and Drug Safety (29), and the database of the Korea Health Industry Development Institute (30). The amino acid database consisted of 19 amino acids. Amino acids were categorized into two types: essential and nonessential. The list of amino acids by type is in Supplementary Table S1. The amino acid database was linked to the recipe database for each FFQ food item. Amino acid intake (g/day) was estimated by multiplying the amino acid content of each food per 1 gram by the amount of food intake. The intake of each type of amino acid was divided into quartiles. Protein intake (g/day) was calculated, based on plant-based protein and animal-based protein, and divided into quartiles.

### 2.3. Definition of dyslipidemia

Blood samples were obtained from individuals who had fasted for at least 8 h and were promptly stored at −80°C until analysis. Serum levels of total TG, total cholesterol (TC), and high-density lipoprotein cholesterol (HDL-C) were measured using an automated analyzer (ADVIA 1650; Bayer Diagnostics, Leverkusen, Germany), based on the standardized protocol. The level of LDL-C was calculated by using the Friedewald formula: LDL-C = TC − (TG/5 + HDL-C). Participants were diagnosed with dyslipidemia if they satisfied one of the following four factors at follow-up (1): hypertriglyceridemia with a blood TG level ≥ 200 mg/dL (2); hypercholesterolemia with a TC level ≥ 240 mg/dL (3); hyper-LDL cholesterolemia with an LDL-C level ≥ 160 mg/dL; or (4) hypo-HDL cholesterolemia with an HDL-C level < 40 mg/dL. The criteria were based on the 2018 Korean guidelines for the management of dyslipidemia (31).

### 2.4. Covariate variables

Sociodemographic and lifestyle variables such as age, sex, education, household income, physical activity, alcohol drinking, and smoking status were obtained from the self-administered questionnaires at baseline. Education level was categorized as less than middle school or beyond high school, and household income was categorized as <2,000,000 Korean Won (KRW)/month [approximately 1,525 United States dollars (USD)/month] or ≥ 2,000,000 KRW/month. Physical activity was defined as the amount of time spent engaging in vigorous physical activity per day and was categorized as inactive (i.e., no physical activity) or active (i.e., >30 min per day). Alcohol drinking status was categorized as nondrinker/past drinker or current drinker. Smoking status was categorized as nonsmoker/past smoker or current smoker.

### 2.5. Statistical analysis

The general characteristics of the study participants were assessed by using the Chi-square test for categorical variables and the generalized linear model for continuous variables. The person-years of each participant were estimated from the enrollment date until the incident date of dyslipidemia and its components for participants with dyslipidemia and its components, or until the date of the most recent follow-up for participants without dyslipidemia and its components. The Cox proportional hazard regression model was used to estimate the hazard ratios (HRs) and 95% confidence intervals (CIs) for dyslipidemia and its components across the quartiles of amino acid and plant-/animal-based protein intake, based on sex.

Multivariable-adjusted analyzes were also conducted. The confounding factors included age, energy intake, body weight, education level, household income, physical activity, alcohol drinking status, and smoking status. In a further analysis, energy-adjusted fat intake obtained from the residual method (32) was adjusted. The *p* value for trend was estimated across median amino acid and plant-/animal-based protein intakes of the quartile groups. All statistical analyzes were conducted using SAS 9.4 (SAS Institute, Cary, NC, United States).

## 3. Results

Among participants without dyslipidemia at baseline, a total of 1,820 (5.1%) cases, 3,828 (10.8%) cases, 2,728 (7.7%) cases, 1,537 (4.3%) cases, and 6,744 (19.0%) cases of incident hypertriglyceridemia, hypercholesterolemia, hyper-LDL cholesterolemia, hypo-HDL cholesterolemia and dyslipidemia, respectively, were reported during a mean follow-up of 5.7 years. Table 1 presents the general characteristics of the study participants without dyslipidemia at baseline (*n* = 35,478), based on the quartile of total amino acid intake. The higher amino acid intake group tended to be younger, drank and smoked more, and had a higher education level (*p* < 0.0001). Compared to the lowest amino acid intake quartile group, the highest amino acid intake quartile group had a higher energy intake and percentage of energy intake from protein and fat, but a lower percentage of energy intake from carbohydrates (*p* < 0.0001).

**Table 1:** Baseline characteristics of the study subjects according to the quartiles of amino acid intake.

	Total amino acid intake (g/day)				
	Q1	Q2	Q3	Q4	*P* value^a^
Total population (*N* = 35,478)	8,869	8,870	8,870	8,869	
Median intake (g/kg/day)	45.9 ± 0.1	62.1 ± 0.1	74.5 ± 0.1	101.8 ± 0.1	
Age (y)	52.1 ± 0.1	52.1 ± 0.1	51.1 ± 0.1	50.3 ± 0.1	<0.0001
Sex (%)					<0.0001
Female	82.0	72.0	69.7	63.5	
Education level (%)^b^					<0.0001
≥ High school	65.0	67.7	72.4	75.6	
Household income (%)^b^					<0.0001
≥2,000,000 KRW/month	60.2	62.4	66.4	66.8	
Physical activity (%)^b^					<0.0001
Active	51.6	53.7	54.1	56.5	
Alcohol drinking status (%)^b^					<0.0001
Current drinker	37.5	42.8	47.1	50.7	
Smoking status (%)^b^					<0.0001
Current smoker	6.8	8.5	9.9	12.4	
Nutrient intake					
Energy intake (kcal/day)	1205.2 ± 3.3	1594.9 ± 3.3	1862.2 ± 3.3	2430.0 ± 3.3	<0.0001
Energy intake from carbohydrate (%E)	74.1 ± 0.1	73.7 ± 0.1	71.1 ± 0.1	66.9 ± 0.1	<0.0001
Energy intake from fat (%E)	12.4 ± 0.1	12.4 ± 0.1	14.4 ± 0.1	17.4 ± 0.1	<0.0001
Energy intake from protein (%E)	12.6 ± 0.0	12.6 ± 0.0	13.5 ± 0.0	15.0 ± 0.0	<0.0001

^a^*p*-values were obtained from the Chi-square test for categorical variables and from the generalized linear model for continuous variables. ^b^Education level is categorized as less than middle school and beyond high school; household income is categorized as <2,000,000 KRW (Korean Won)/month and ≥ 2,000,000 KRW/month; physical activity is defined as the time of engaging in vigorous physical activity per day: inactive (i.e., no physical activity) or active (i.e., >30 min per day); alcohol drinking status is categorized as nondrinker/past drinker and current drinker; and smoking status is categorized as nonsmoker/past smoker and current smoker.

Q1–4, quartile 1–4.

**Figure 2:**
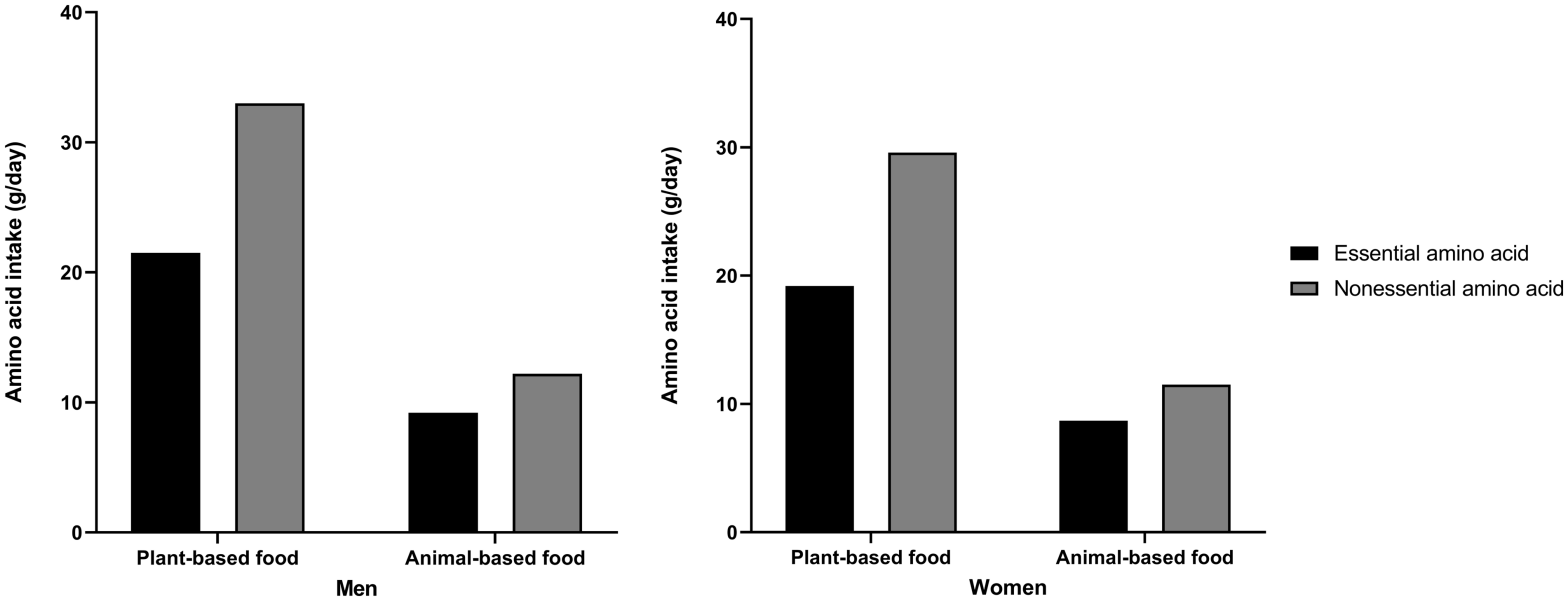
Essential and nonessential amino acid intake from plant- and animal-based food sources.

The intakes of essential amino acids and nonessential amino acids from plant- and animal-based food sources in Korean adults are presented in Figure 2. Korean adults had higher essential and nonessential amino acid intake from plant-based foods (men: essential 21.5 ± 5.3 g/day, nonessential 33.0 ± 8.4 g/day; women: essential 19.2 ± 5.7 g/day, nonessential 29.6 ± 8.8 g/day) than from animal-based foods (men: essential 9.2 ± 6.3 g/day, nonessential 12.2 ± 8.4 g/day; women: essential 8.7 ± 6.3 g/day, nonessential 11.5 ± 8.3 g/day). In addition, nonessential amino acid intake was higher than essential amino acid intake from plant- and animal-based food sources. The top 10 food sources contributing to each type of amino acid intake in Korean adults are described in Table 2. Overall, whole grain mixed rice, white rice, red meat, fish, soybean mixed rice, dairy, noodles, legumes, eggs, white meat, and bread were the major food groups for essential and nonessential amino acid intake. Whole grain mixed rice was the food group that contributed the most to the intake of essential and nonessential amino acids in men (34.9 and 33.4%, respectively) and in women (38.4 and 36.8%, respectively). Koreans obtained nearly one-half of amino acids from plant-based foods, whole grain mixed rice, and white rice (men: essential 51.3%, nonessential 48.9%; women: essential 48.4%, nonessential 46.2%). Detailed information of major food groups consumed by Korean adults is described in Supplementary Table S2.

**Table 2:** Top ten food sources contributing to amino acid intake.

Men				
Rank	Food group	Contribution (%)	*Cum* (%)	Intake (g/day)
Essential amino acids				
1	Whole grain mixed rice	34.9	34.9	10.4 ± 8.6
2	White rice	16.4	51.3	4.8 ± 7.7
3	Red meat	10.1	61.4	3.4 ± 3.4
4	Fish	7.5	68.9	2.4 ± 2.3
5	Soybean mixed rice	4.4	73.3	1.3 ± 4.9
6	Dairy	3.8	77.2	1.2 ± 1.5
7	Legumes	3.4	80.5	1.1 ± 1.0
8	Noodles	3.2	83.7	1.0 ± 1.2
9	Eggs	2.1	85.9	0.7 ± 0.9
10	White meat	2.0	87.8	0.6 ± 0.9
Nonessential amino acids				
1	Whole grain mixed rice	33.4	33.4	14.7 ± 12.1
2	White rice	15.5	48.9	6.6 ± 10.5
3	Red meat	9.3	58.1	4.5 ± 4.6
4	Fish	6.5	64.6	3.1 ± 2.9
5	Noodles	5.1	69.7	2.4 ± 2.7
6	Soybean mixed rice	4.2	74.0	1.9 ± 6.8
7	Legumes	3.8	77.8	1.8 ± 1.6
8	Dairy	3.5	81.3	1.6 ± 2.1
9	Eggs	1.9	83.2	0.9 ± 1.1
10	White meat	1.8	85.0	0.9 ± 1.3
Women				
Essential amino acids				
1	Whole grain mixed rice	38.4	38.4	10.3 ± 7.4
2	White rice	10.0	48.4	2.7 ± 5.8
3	Fish	8.6	57.0	2.5 ± 2.5
4	Red meat	8.4	65.4	2.6 ± 3.0
5	Dairy	5.6	71.0	1.6 ± 1.8
6	Soybean mixed rice	4.7	75.7	1.3 ± 4.6
7	Legumes	3.9	79.6	1.1 ± 1.1
8	Eggs	2.4	82.0	0.7 ± 0.8
9	Noodles	2.3	84.4	0.7 ± 0.9
10	White meat	1.9	86.3	0.6 ± 0.9
Nonessential amino acids				
1	Whole grain mixed rice	36.8	36.8	14.6 ± 10.5
2	White rice	9.4	46.2	3.6 ± 7.9
3	Red meat	7.7	53.9	3.4 ± 4.0
4	Fish	7.5	61.3	3.2 ± 3.2
5	Dairy	5.0	66.4	2.1 ± 2.4
6	Soybean mixed rice	4.6	70.9	1.9 ± 6.4
7	Legumes	4.4	75.4	1.9 ± 1.9
8	Noodles	3.7	79.1	1.6 ± 2.2
9	Bread	2.3	81.3	1.0 ± 1.6
10	Eggs	2.1	83.4	0.9 ± 1.1

Cum: cumulative contribution.

Table 3 shows the association between amino acid intake and dyslipidemia and its components. Essential and nonessential amino acid intake showed inverse relationships with the incidence of dyslipidemia and its components in men and women. Compared to the lowest group, the highest quartile for essential amino acid intake had a 22 and 14% decreased risk of dyslipidemia in men (HR = 0.78; 95% CI, 0.63–0.97; *P* for trend = 0.0088) and in women (HR = 0.86; 95% CI, 0.76–0.99; *P* for trend = 0.0201), respectively. In addition, essential amino acid intake had a negative association with hypercholesterolemia (HR = 0.66; 95% CI, 0.46–0.95; *P* for trend = 0.0157) and hyper-LDL cholesterolemia (HR = 0.69; 95% CI, 0.46–1.02; *P* for trend = 0.0436) in men.

**Table 3:** Hazard ratios and 95% confidence intervals for the incidence of dyslipidemia and its components, based on the amino acid intake quartile^a^.

	Amino acid intake (g/day)				
	Q1	Q2	Q3	Q4	*P* for trend
Men					
Essential amino acid					
Hypertriglyceridemia	Ref	1.08 (0.86–1.35)	0.94 (0.72–1.21)	0.92 (0.65–1.31)	0.4645
Hypercholesterolemia	Ref	0.90 (0.72–1.12)	0.75 (0.58–0.97)	0.66 (0.46–0.95)	0.0157
Hyper-LDL cholesterolemia	Ref	0.88 (0.68–1.13)	0.69 (0.52–0.93)	0.69 (0.46–1.02)	0.0436
Hypo-HDL cholesterolemia	Ref	1.01 (0.80–1.26)	0.90 (0.70–1.16)	0.73 (0.51–1.04)	0.0563
Dyslipidemia	Ref	1.00 (0.88–1.15)	0.86 (0.74–1.01)	0.78 (0.63–0.97)	0.0088
Nonessential amino acid					
Hypertriglyceridemia	Ref	1.10 (0.87–1.39)	0.97 (0.75–1.26)	0.91 (0.63–1.32)	0.4409
Hypercholesterolemia	Ref	0.81 (0.65–1.02)	0.72 (0.56–0.93)	0.57 (0.39–0.83)	0.0035
Hyper-LDL cholesterolemia	Ref	0.84 (0.65–1.08)	0.65 (0.48–0.87)	0.60 (0.40–0.90)	0.0082
Hypo-HDL cholesterolemia	Ref	0.95 (0.76–1.20)	0.98 (0.76–1.27)	0.80 (0.56–1.15)	0.2617
Dyslipidemia	Ref	0.96 (0.84–1.10)	0.87 (0.75–1.02)	0.75 (0.60–0.94)	0.0069
Women					
Essential amino acid					
Hypertriglyceridemia	Ref	0.98 (0.82–1.18)	0.88 (0.72–1.08)	0.79 (0.60–1.04)	0.0688
Hypercholesterolemia	Ref	0.98 (0.88–1.09)	0.88 (0.78–0.99)	0.90 (0.77–1.07)	0.1448
Hyper-LDL cholesterolemia	Ref	0.99 (0.87–1.13)	0.90 (0.78–1.05)	0.93 (0.76–1.13)	0.3370
Hypo-HDL cholesterolemia	Ref	0.83 (0.67–1.03)	0.91 (0.72–1.15)	0.75 (0.55–1.02)	0.1207
Dyslipidemia	Ref	0.96 (0.88–1.04)	0.89 (0.80–0.98)	0.86 (0.76–0.99)	0.0201
Nonessential amino acid					
Hypertriglyceridemia	Ref	0.99 (0.83–1.18)	0.87 (0.71–1.08)	0.81 (0.61–1.08)	0.1038
Hypercholesterolemia	Ref	0.92 (0.82–1.02)	0.88 (0.77–0.99)	0.83 (0.70–0.99)	0.0349
Hyper-LDL cholesterolemia	Ref	0.92 (0.81–1.05)	0.92 (0.79–1.06)	0.87 (0.71–1.07)	0.2111
Hypo-HDL cholesterolemia	Ref	0.80 (0.64–0.99)	0.85 (0.68–1.08)	0.70 (0.51–0.96)	0.0510
Dyslipidemia	Ref	0.90 (0.83–0.98)	0.87 (0.78–0.96)	0.81 (0.71–0.93)	0.0024

^a^All values have been adjusted for age, energy intake, body weight, education level, household income, physical activity, alcohol drinking status, and smoking status.

Q1–4, quartile 1–4; LDL, low-density lipoprotein; HDL, high-density lipoprotein; Ref, reference.

The intake of nonessential amino acids was also inversely associated with the incidence of dyslipidemia in men and women. Compared to the lowest nonessential amino acid intake quartile group, men in the highest quartile group had a 25% reduced risk of dyslipidemia (HR = 0.75; 95% CI, 0.60–0.94; *P* for trend = 0.0069) and women had a 19% reduced risk of dyslipidemia (HR = 0.81; 95% CI, 0.71–0.93; *P* for trend = 0.0024). Furthermore, in men, the nonessential amino acid intake was negatively associated with hypercholesterolemia (HR = 0.57; 95% CI, 0.39–0.83; *P* for trend = 0.0035) and hyper-LDL cholesterolemia (HR = 0.60; 95% CI, 0.40–0.90; *P* for trend = 0.0082). In women, nonessential amino acid intake was also associated with a decreased risk of hypercholesterolemia (HR = 0.83; 95% CI, 0.70–0.99; *P* for trend = 0.0349) and hypo-HDL cholesterolemia (HR = 0.70; 95% CI, 0.51–0.96; *P* for trend = 0.0510). The median intake of amino acids and number of cases and person-years for dyslipidemia and its components, based on the quartile group, are shown in Supplementary Tables S3, S4, respectively.

To further investigate whether the protein food source affects the incidence of dyslipidemia, the association of plant- and animal-based protein intake with dyslipidemia and its components was assessed (Table 4). The median intake of protein and the person-years and number of cases of dyslipidemia and its components, based on quartile group, are shown in Supplementary Tables S3, S5, respectively. As a result, plant-based protein intake had a negative relationship with dyslipidemia in men and in women. The highest quartile group of plant-based protein intake had a 19 and 21% reduced risk of dyslipidemia in men (HR = 0.81; 95% CI, 0.67–0.98; *P* for trend = 0.0157) and in women (HR = 0.79; 95% CI, 0.70–0.89; *P* for trend = 0.0001), respectively, compared to the lowest group. Plant-based protein intake was also inversely associated with hypercholesterolemia and hyper-LDL cholesterolemia in men and women. Contrary to this finding, in women, animal-based protein intake had a positive relationship with hypercholesterolemia (HR = 1.24; 95% CI, 1.11–1.40; *P* for trend = 0.0003) and with dyslipidemia (HR = 1.12, 95% CI, 1.02–1.24; *P* for trend = 0.0173). However, this positive association disappeared after further adjusting for energy-adjusted fat intake, as shown in Supplementary Table S6. In addition, the essential and nonessential amino acid intake with dyslipidemia had a stronger negative relationship, after further adjustment for energy-adjusted fat intake, as shown in Supplementary Table S7.

**Table 4:** Hazard ratios and 95% confidence intervals for the incidence of dyslipidemia and its components, based on the protein intake quartile^a^.

	Protein intake (g/day)				
	Q1	Q2	Q3	Q4	*P* for trend
Men					
Plant-based protein					
Hypertriglyceridemia	Ref	1.10 (0.88–1.37)	0.93 (0.73–1.19)	1.03 (0.75–1.40)	0.9743
Hypercholesterolemia	Ref	0.94 (0.76–1.17)	0.83 (0.65–1.05)	0.62 (0.44–0.86)	0.0031
Hyper-LDL cholesterolemia	Ref	0.97 (0.76–1.24)	0.78 (0.59–1.02)	0.59 (0.41–0.85)	0.0027
Hypo-HDL cholesterolemia	Ref	1.06 (0.85–1.33)	1.00 (0.78–1.26)	0.88 (0.64–1.20)	0.3306
Dyslipidemia	Ref	1.01 (0.88–1.15)	0.90 (0.78–1.04)	0.81 (0.67–0.98)	0.0157
Animal-based protein					
Hypertriglyceridemia	Ref	1.05 (0.84–1.31)	1.09 (0.87–1.37)	0.98 (0.76–1.27)	0.7765
Hypercholesterolemia	Ref	1.06 (0.85–1.31)	1.06 (0.84–1.34)	1.14 (0.88–1.48)	0.3472
Hyper-LDL cholesterolemia	Ref	1.01 (0.78–1.29)	1.09 (0.84–1.41)	1.09 (0.81–1.45)	0.5322
Hypo-HDL cholesterolemia	Ref	1.07 (0.87–1.33)	0.97 (0.77–1.21)	1.05 (0.82–1.35)	0.8499
Dyslipidemia	Ref	1.05 (0.92–1.20)	1.05 (0.92–1.21)	1.06 (0.91–1.24)	0.5190
Women					
Plant-based protein					
Hypertriglyceridemia	Ref	0.88 (0.73–1.05)	0.90 (0.74–1.10)	0.82 (0.64–1.05)	0.1331
Hypercholesterolemia	Ref	0.85 (0.76–0.94)	0.81 (0.73–0.91)	0.72 (0.62–0.84)	<0.0001
Hyper-LDL cholesterolemia	Ref	0.86 (0.76–0.98)	0.86 (0.74–0.98)	0.84 (0.70–1.00)	0.0299
Hypo-HDL cholesterolemia	Ref	1.06 (0.85–1.32)	1.09 (0.86–1.38)	1.06 (0.80–1.42)	0.6244
Dyslipidemia	Ref	0.88 (0.80–0.95)	0.86 (0.79–0.95)	0.79 (0.70–0.89)	0.0001
Animal-based protein					
Hypertriglyceridemia	Ref	1.02 (0.86–1.20)	1.01 (0.85–1.20)	1.00 (0.82–1.22)	0.9488
Hypercholesterolemia	Ref	1.10 (0.99–1.22)	1.18 (1.06–1.31)	1.24 (1.11–1.40)	0.0003
Hyper-LDL cholesterolemia	Ref	1.11 (0.98–1.25)	1.19 (1.05–1.35)	1.15 (1.00–1.33)	0.0716
Hypo-HDL cholesterolemia	Ref	1.10 (0.91–1.33)	0.99 (0.81–1.22)	0.93 (0.74–1.17)	0.3505
Dyslipidemia	Ref	1.06 (0.97–1.15)	1.10 (1.01–1.20)	1.12 (1.02–1.24)	0.0173

^a^All values have been adjusted for age, energy intake, body weight, education level, household income, physical activity, alcohol drinking status, and smoking status.

Q1–4, quartile 1–4; LDL, low-density lipoprotein; HDL, high-density lipoprotein; Ref, reference.

The association of amino acid and protein intake for each participant’s body weight with dyslipidemia was also assessed to evaluate the appropriate intake level with respect to physical condition. The results are presented in Supplementary Tables S8, S9. All types of amino acid intake were inversely associated with all components of dyslipidemia incidence in men and women. Plant-based protein intake was also negatively associated with all components of dyslipidemia incidence in men and women. Animal-based protein intake was negatively associated with hypercholesterolemia, hypo-HDL cholesterolemia, and dyslipidemia in men, and with hypertriglyceridemia, hypercholesterolemia, hyper-LDL cholesterolemia, and dyslipidemia in women.

## 4. Discussion

The purpose of this study was to investigate whether the incidence of dyslipidemia in Korean adults would be associated with the consumption of essential and nonessential amino acids, as well as with the protein food sources (i.e., plant or animal) of these amino acids. We found that essential and nonessential amino acid intake was associated with a decreased risk of the incidence dyslipidemia and its parameters in this prospective cohort study of Korean adults ≥ 40 years old. Koreans primarily had essential and nonessential amino acid intakes from plant-based foods, whole grain mixed rice, and white rice, which accounted for approximately one-half of the amino acid intake. Thus, plant-based protein intake also had a negative relationship with dyslipidemia, whereas animal-based protein intake had a positive relationship with dyslipidemia. However, plant-based protein intake had a stronger negative association and animal-based protein intake had a nonsignificant association with dyslipidemia, after adjusting for energy-adjusted fat intake. The essential and nonessential amino acid intake also had stronger negative associations with dyslipidemia after adjusting for energy-adjusted fat intake. Amino acid intake may have preventive effects on dyslipidemia, independently of the protein food sources.

Although the mechanisms underlying the relationship between dietary amino acids and metabolic disorders are unclear, a few studies have demonstrated that essential amino acid supplementation lowers plasma levels of TG, LDL, and TC in humans (33, 34). Reports have indicated that various amino acid patterns are implicated in the metabolism related to nonalcoholic fatty liver disease. Synthesis and catabolism of protein and amino acids are involved in metabolic pathways in the liver such as β-oxidation and tricarboxylic acid and have a role in oxidative stress and inflammation. In individuals with nonalcoholic fatty liver disease, investigators have observed increased levels of several essential amino acids such as the branched-chain amino acids such as isoleucine, leucine, and valine and the aromatic amino acids, phenylalanine and tryptophan, and decreased levels of the nonessential amino acids, tyrosine, glutamine, serine, and glycine (35).

Another possibility that other dietary factors may influence amino acid metabolism related to dyslipidemia has been observed in other epidemiological studies (36–40). Higher intake of essential amino acids and better nutrient adequacy have been associated with decreased all-cause and cardiovascular disease mortality in United States adults (36). Contrary to this finding, an amino acid intake dietary pattern, which was highly correlated with essential amino acids such as lysine, methionine, histidine, threonine, and branched-chain amino acids, and meat protein was associated with increased cardiovascular mortality in a prospective study of adults in the United States and Canada (37). In addition, the intake of animal-based protein has been positively associated with metabolic diseases, including dyslipidemia and mortality (38–40). The available evidence suggests that undisclosed effects may exist that result from interactions between amino acids and other dietary components, depending on the food sources. Therefore, further research, including structural studies, aimed at examining the relationship between amino acid intake, other food components, and metabolic diseases is needed.

To investigate whether protein food sources and other nutrients affect dyslipidemia, we conducted further analyzes of the association of protein intake by food source and other nutrient intake with dyslipidemia in this study. The results indicated that plant-based protein intake was inversely associated with dyslipidemia, whereas animal-based protein intake was positively associated with dyslipidemia. Nevertheless, we believe that the association was influenced by fat intake because nonsignificant correlations with animal-based protein intake were observed, after adjusting for energy-adjusted fat intake. These findings indicate that amino acids may have an impact on dyslipidemia, regardless of the food source. Furthermore, we hypothesize that food components may interact with amino acids or participate in amino acid metabolism because the inverse relationship between essential and nonessential amino acid intake and dyslipidemia became stronger after adjusting for energy-adjusted fat intake. Future research should aim to investigate the correlation between amino acid intake and other dietary components beyond fat.

Furthermore, the findings also confirmed that appropriate amino acid and protein intake may be helpful for the prevention of dyslipidemia, independently of protein food sources. As shown in Supplementary Tables S8, S9, all types of amino acid and plant-based protein intake per each participant’s body weight were inversely associated with all components of dyslipidemia incidence in men and in women. Animal-based protein intake was negatively associated with several dyslipidemia components in women. These findings imply that the effect of amino acid and protein intake on cardiometabolic function should be evaluated by considering the absolute amount of intake and the protein requirements, based on body weight.

To the best of our knowledge, this study is the first prospective study to examine the relationship between amino acid intake and the likelihood of developing dyslipidemia among Korean adults. However, this study has several limitations.

First, amino acid intake may have been underestimated because the FFQ and the amino acid database did not obtain information on amino acid intake from all food and supplements consumed by the participants. This limitation is an impetus for future studies to develop a detailed amino acid database.

Second, the estimation of amino acid and protein food sources may have been biased because of the food items on the FFQ. The number of plant-based food sources are much higher than that of animal-based food sources (Supplementary Table S2). Moreover, several food lists, for example, pizza/hamburger and jam/honey/butter/margarine could not be individually divided into plant- or animal-based foods. These limitations may induce high amino acid and protein intake from plant-based foods. In addition, cumulative and long-term dietary status and lifestyle factors could not be assessed because we used only baseline data. Thus, changes in diet or lifestyle during the follow-up period were not considered. Although the Korean diet has a higher proportion of plant-based protein than animal-based protein (13) and a rice-based dietary pattern, animal-based protein intake has recently increased since the time the survey of this present study was conducted (41). The disparity in animal-based protein intake data obtained from previous and recent studies may result in inconclusive associations.

Third, although we adjusted for potential confounders, unrevealed or residual confounders may have affected the relationship between amino acid intake and dyslipidemia.

Finally, generalizing the study findings from middle-aged participants to all generations and populations is difficult. Despite these limitations, the findings of this study can offer evidence for suitable amino acid and protein intake when creating dietary guidelines for cardiovascular health.

## 5. Conclusion

In conclusion, the intake of all types of amino acids, including essential and nonessential amino acids, was inversely associated with the incidence of dyslipidemia and its components in the Korean adults aged ≥40 years, independently of plant- and animal-based protein food sources. Approximately one-half of essential and nonessential amino acids was obtained from plant-based foods, and plant-based protein intake was negatively associated with the incidence of dyslipidemia. Animal-based protein intake was not significantly associated with dyslipidemia and essential and nonessential amino acid intake showed stronger negative associations with dyslipidemia, after adjusting for energy-adjusted fat intake. Our study findings suggest that amino acid intake may be beneficial in the management of dyslipidemia and can offer evidence for the appropriate amount of amino acid and plant- and animal-based protein intake for cardiovascular health in Korean adults.

## Data availability statement

The original contributions presented in the study are included in the article/Supplementary material, further inquiries can be directed to the corresponding authors.

## Ethics statement

The studies involving human participants were reviewed and approved by Chung-Ang University (Ansung, Republic of Korea; approval no., 1041078-202109-HR-278-01). The patients/participants provided their written informed consent to participate in this study.

## Author contributions

SC, KH, and SS designed the study. SC analyzed the data and wrote the first draft of the manuscript. SC, KH, and HJ participated in interpreted the results. JHP, HJ, and SS reviewed and critically edited the manuscript. All authors read and approved the final manuscript.

## Funding

This work was supported by a National Research Foundation of Korea (NRF) (grant nos. 2021R1G1A1008495 and 2022R1F1A1074279) and a Korea Food Research Institute (grant no. E0210601-02) funded by the Ministry of Science and Information and Communication Technology (Sejong, Republic of Korea).

## Conflict of interest

The authors declare that the research was conducted in the absence of any commercial or financial relationships that could be construed as a potential conflict of interest.

## Publisher’s note

All claims expressed in this article are solely those of the authors and do not necessarily represent those of their affiliated organizations, or those of the publisher, the editors and the reviewers. Any product that may be evaluated in this article, or claim that may be made by its manufacturer, is not guaranteed or endorsed by the publisher.

## Abbreviations

CI: confidence interval
CVD: cardiovascular disease
FFQ: Food Frequency Questionnaire
HEXA: Health Examinee
HR: hazard ratio
KoGES: Korean Genome and Epidemiology Study
TC: total cholesterol
TG: triglyceride
