# Supplementary material for: Amino acid intake with protein food source and incident dyslipidemia in Korean adults from the Ansan and Ansung Study and the Health Examinee Study
Source: Front Nutr. 2023 Jul 21;10:1195349. doi: 10.3389/fnut.2023.1195349 (PMC10401580; doi:10.3389/fnut.2023.1195349)
Supplement: Supplementary file 1 [file Data_Sheet_1.docx]

1. ***Supplementary Material***

**Supplementary Table 1. Amino acids included in the database of the study**

|  | **Amino acid list** |
| --- | --- |
| **Essential amino acids** | Isoleucine, leucine, valine, lysine, methionine, phenylalanine, threonine, tryptophan, histidine, and arginine |
| **Nonessential amino acids** | Tyrosine, cysteine, alanine, aspartic acid, glutamic acid, glycine, proline, serine, and taurine |

**Supplementary Table 2. Major food groups consumed by Korean adults participating in the Ansan and Ansung Study and the Health Examinee Study**

| **Food group** | **Food list** |
| --- | --- |
| **Plant-based food** |  |
| White rice | Cooked rice |
| Whole grain mixed rice | Cooked rice with other grains |
| Soybean mixed rice | Cooked rice with soybean |
| Other grains | Cereals, corn flakes, parched cereal powder |
| Rice cake | Rice cake, rice cake with soup, other rice cakes |
| Noodles | Ramen, wheat noodles with soup, Chinese noodles, buckwheat noodles |
| Dumplings | Dumpling, dumpling with soup |
| Bread | Loaf bread, sandwich, toast, other bread, pizza, hamburger |
| Potatoes | Potatoes, sweet potatoes, starch jelly, starch vermicelli |
| Legumes | Legumes, soybean paste, soup and stew with soybean paste, tofu, soybean milk |
| Seaweed | Laver, kelp, sea mustard |
| Vegetables | Radish, Korean cabbages, spinach, lettuce, perilla leaf, other green vegetables, deoduck/doraji (types of white root), bean sprouts, mushrooms, cucumber, carrot, onion, green pepper, pumpkin, all kimchi, Korean-style pickles, and others |
| Fruits | Strawberry, melon, watermelon, peach, plum, banana, persimmon, tangerine, pear, apple, orange, grape, tomato, and others |
| Nuts | Peanuts, almonds, pine nuts |
| Sugar and fat | Candies, chocolates, cakes, cookies, crackers, jam/honey/butter/margarine with bread |
| Beverages | Coffee, coffee sugar, coffee cream, green tea, soft drinks, other drinks |
| **Animal-based food** |  |
| Red meat | Pork belly, roasted pork, braised pork, steak, roasted beef, beef soup, edible viscera |
| White meat | Fried chicken, chicken stew |
| Processed meat | Ham, sausage |
| Fish | Raw fish, mackerel, Pacific saury, Spanish mackerel, Hair tail, eel, yellow croaker, sea bream, flat fish, Alaska pollack, anchovy, salt-fermented fish |
| Seafood | Cuttlefish, octopus, clam, oyster, crab, and shrimp |
| Processed fish | Canned tuna, fish paste |
| Dairy | Milk, yogurt, ice cream, cheese |
| Eggs | Eggs |

**Supplementary Table 3. Median intake, based on the amino acid type and protein intake quartile**

|  | **Q1** | **Q2** | **Q3** | **Q4** |
| --- | --- | --- | --- | --- |
| **Men** |  |  |  |  |
| **Amino acid (g/day)** |  |  |  |  |
| Essential amino acid | 21.8±0.1 | 26.9±0.0 | 31.9±0.0 | 40.7±0.2 |
| Nonessential amino acid | 31.9±0.1 | 39.5±0.0 | 47.0±0.1 | 59.9±0.2 |
| **Protein (g/day)** |  |  |  |  |
| Plant-based protein | 45.7±0.2 | 55.0±0.0 | 61.3±0.0 | 76.0±0.2 |
| Animal-based protein | 8.9±0.1 | 16.3±0.0 | 24.4±0.1 | 40.3±0.4 |
|  |  |  |  |  |
| **Women** |  |  |  |  |
| **Amino acid (g/day)** |  |  |  |  |
| Essential amino acid | 18.5±0.0 | 24.5±0.0 | 29.3±0.0 | 37.5±0.1 |
| Nonessential amino acid | 27.3±0.1 | 36.0±0.0 | 43.0±0.0 | 55.3±0.2 |
| **Protein (g/day)** |  |  |  |  |
| Plant-based protein | 35.3±0.1 | 50.3±0.0 | 57.5±0.0 | 69.1±0.2 |
| Animal-based protein | 8.3±0.0 | 15.5±0.0 | 23.1±0.0 | 37.9±0.2 |

*Q1–4*, quartile 1–4

**Supplementary Table 4. Person-years and number of cases for the incidence of dyslipidemia and its components, based on the amino acid intake quartile**

|  |  | **Amino acid intake (g/day)** | | | |
| --- | --- | --- | --- | --- | --- |
|  |  | **Q1** | **Q2** | **Q3** | **Q4** |
| **Men (total=10,012)** | N | 2,503 | 2,503 | 2,503 | 2,503 |
| **Essential amino acid** | Total person-years | 14,097 | 14,451 | 15,059 | 15,565 |
|  | Number of cases |  |  |  |  |
|  | Hypertriglyceridemia | 152 | 183 | 177 | 193 |
|  | Hypercholesterolemia | 184 | 177 | 165 | 154 |
|  | Hyper-LDL cholesterolemia | 143 | 135 | 121 | 134 |
|  | Hypo-HDL cholesterolemia | 163 | 183 | 186 | 181 |
|  | Dyslipidemia | 452 | 500 | 480 | 492 |
| **Nonessential amino acid** | Total person-years | 14,138 | 14,380 | 15,004 | 15,650 |
|  | Number of cases |  |  |  |  |
|  | Hypertriglyceridemia | 151 | 184 | 179 | 191 |
|  | Hypercholesterolemia | 191 | 168 | 171 | 150 |
|  | Hyper-LDL cholesterolemia | 147 | 134 | 121 | 131 |
|  | Hypo-HDL cholesterolemia | 166 | 170 | 190 | 187 |
|  | Dyslipidemia | 461 | 484 | 489 | 490 |
|  |  |  |  |  |  |
| **Women (total=25,466)** | N | 6,366 | 6,367 | 6,367 | 6,366 |
| **Essential amino acid** | Total person-years | 34,328 | 34,734 | 36,131 | 38,059 |
|  | Number of cases |  |  |  |  |
|  | Hypertriglyceridemia | 282 | 290 | 278 | 265 |
|  | Hypercholesterolemia | 825 | 797 | 748 | 778 |
|  | Hyper-LDL cholesterolemia | 553 | 551 | 532 | 559 |
|  | Hypo-HDL cholesterolemia | 187 | 183 | 226 | 228 |
|  | Dyslipidemia | 1,219 | 1,198 | 1,184 | 1,219 |
| **Nonessential amino acid** | Total person-years | 34,282 | 34,674 | 35,904 | 38,393 |
|  | Number of cases |  |  |  |  |
|  | Hypertriglyceridemia | 281 | 291 | 270 | 273 |
|  | Hypercholesterolemia | 835 | 771 | 769 | 773 |
|  | Hyper-LDL cholesterolemia | 559 | 528 | 550 | 558 |
|  | Hypo-HDL cholesterolemia | 192 | 182 | 217 | 233 |
|  | Dyslipidemia | 1,238 | 1,167 | 1,189 | 1,226 |

*Q1–4*, quartile 1–4; *LDL*, low-density lipoprotein; *HDL* high-density lipoprotein

**Supplementary Table 5. Person-years and number of cases for the incidence of dyslipidemia and its components, based on the protein intake quartile**

|  |  | **Protein intake (g/day)** | | | |
| --- | --- | --- | --- | --- | --- |
|  |  | **Q1** | **Q2** | **Q3** | **Q4** |
| **Men (total=10,012)** | N | 2,503 | 2,503 | 2,503 | 2,503 |
| **Plant-based protein** | Total person-years | 13,965 | 14,203 | 15,187 | 15,817 |
|  | Number of cases |  |  |  |  |
|  | Hypertriglyceridemia | 157 | 179 | 169 | 200 |
|  | Hypercholesterolemia | 183 | 177 | 179 | 141 |
|  | Hyper-LDL cholesterolemia | 139 | 142 | 134 | 118 |
|  | Hypo-HDL cholesterolemia | 158 | 170 | 188 | 197 |
|  | Dyslipidemia | 458 | 478 | 491 | 497 |
| **Animal-based protein** | Total person-years | 14,952 | 14,464 | 14,551 | 15,205 |
|  | Number of cases |  |  |  |  |
|  | Hypertriglyceridemia | 159 | 173 | 191 | 182 |
|  | Hypercholesterolemia | 167 | 169 | 169 | 175 |
|  | Hyper-LDL cholesterolemia | 130 | 126 | 138 | 139 |
|  | Hypo-HDL cholesterolemia | 186 | 179 | 162 | 186 |
|  | Dyslipidemia | 467 | 472 | 481 | 504 |
|  |  |  |  |  |  |
| **Women (total=25,466)** | N | 6,366 | 6,367 | 6,367 | 6,366 |
| **Plant-based protein** | Total person-years | 33,611 | 35,149 | 35,617 | 38,876 |
|  | Number of cases |  |  |  |  |
|  | Hypertriglyceridemia | 280 | 271 | 276 | 288 |
|  | Hypercholesterolemia | 866 | 770 | 753 | 759 |
|  | Hyper-LDL cholesterolemia | 578 | 518 | 523 | 576 |
|  | Hypo-HDL cholesterolemia | 154 | 201 | 209 | 260 |
|  | Dyslipidemia | 1,233 | 1,169 | 1,172 | 1,246 |
| **Animal-based protein** | Total person-years | 36,573 | 35,035 | 35,030 | 36,615 |
|  | Number of cases |  |  |  |  |
|  | Hypertriglyceridemia | 308 | 277 | 266 | 264 |
|  | Hypercholesterolemia | 759 | 774 | 794 | 821 |
|  | Hyper-LDL cholesterolemia | 527 | 546 | 569 | 553 |
|  | Hypo-HDL cholesterolemia | 223 | 209 | 191 | 201 |
|  | Dyslipidemia | 1,221 | 1,177 | 1,192 | 1,230 |

*Q1–4,* quartile 1–4; *LDL*, low-density lipoprotein; *HDL* high-density lipoprotein

**Supplementary Table 6. Hazard ratios and 95% confidence intervals, further adjusted for energy-adjusted fat intake for the incidence of dyslipidemia and its components, based on the protein intake quartile** ^a^

|  | **Protein intake (g/day)** | | | |  |
| --- | --- | --- | --- | --- | --- |
|  | **Q1** | **Q2** | **Q3** | **Q4** | ***P* for trend** |
| **Men** |  |  |  |  |  |
| **Plant-based protein** |  |  |  |  |  |
| Hypertriglyceridemia | Ref | 1.10 (0.87-1.38) | 0.93 (0.72-1.19) | 1.02 (0.73-1.42) | 0.9139 |
| Hypercholesterolemia | Ref | 0.96 (0.77-1.20) | 0.85 (0.66-1.08) | 0.64 (0.46-0.91) | 0.0079 |
| Hyper-LDL cholesterolemia | Ref | 0.97 (0.75-1.24) | 0.77 (0.58-1.02) | 0.58 (0.40-0.86) | 0.0029 |
| Hypo-HDL cholesterolemia | Ref | 1.05 (0.83-1.33) | 0.98 (0.77-1.26) | 0.86 (0.62-1.19) | 0.2782 |
| Dyslipidemia | Ref | 1.01 (0.88-1.16) | 0.89 (0.77-1.04) | 0.81 (0.66-0.98) | 0.0162 |
| **Animal-based protein** |  |  |  |  |  |
| Hypertriglyceridemia | Ref | 1.06 (0.84-1.34) | 1.11 (0.86-1.44) | 1.01 (0.72-1.40) | 0.8653 |
| Hypercholesterolemia | Ref | 1.01 (0.80-1.28) | 0.99 (0.76-1.29) | 1.01 (0.72-1.41) | 0.9688 |
| Hyper-LDL cholesterolemia | Ref | 1.00 (0.77-1.30) | 1.07 (0.80-1.44) | 1.06 (0.73-1.55) | 0.7085 |
| Hypo-HDL cholesterolemia | Ref | 1.10 (0.87-1.37) | 1.00 (0.77-1.29) | 1.10 (0.80-1.52) | 0.6810 |
| Dyslipidemia | Ref | 1.06 (0.92-1.21) | 1.06 (0.90-1.24) | 1.07 (0.88-1.31) | 0.6094 |
|  |  |  |  |  |  |
| **Women** |  |  |  |  |  |
| **Plant-based protein** |  |  |  |  |  |
| Hypertriglyceridemia | Ref | 0.87 (0.71-1.05) | 0.89 (0.72-1.10) | 0.81 (0.62-1.06) | 0.1524 |
| Hypercholesterolemia | Ref | 0.89 (0.79-1.00) | 0.86 (0.76-0.98) | 0.77 (0.66-0.91) | 0.0018 |
| Hyper-LDL cholesterolemia | Ref | 0.87 (0.76-1.00) | 0.87 (0.74-1.01) | 0.85 (0.70-1.03) | 0.0907 |
| Hypo-HDL cholesterolemia | Ref | 0.97 (0.76-1.23) | 0.98 (0.76-1.27) | 0.94 (0.69-1.29) | 0.7432 |
| Dyslipidemia | Ref | 0.89 (0.81-0.98) | 0.88 (0.80-0.98) | 0.81 (0.71-0.93) | 0.0022 |
| **Animal-based protein** |  |  |  |  |  |
| Hypertriglyceridemia | Ref | 1.00 (0.84-1.19) | 0.98 (0.80-1.20) | 0.95 (0.74-1.22) | 0.6416 |
| Hypercholesterolemia | Ref | 1.07 (0.96-1.19) | 1.12 (1.00-1.26) | 1.15 (0.99-1.33) | 0.0986 |
| Hyper-LDL cholesterolemia | Ref | 1.11 (0.97-1.26) | 1.19 (1.03-1.37) | 1.14 (0.95-1.36) | 0.2700 |
| Hypo-HDL cholesterolemia | Ref | 1.18 (0.96-1.44) | 1.11 (0.88-1.41) | 1.13 (0.84-1.52) | 0.6623 |
| Dyslipidemia | Ref | 1.04 (0.95-1.13) | 1.07 (0.97-1.18) | 1.07 (0.95-1.21) | 0.3129 |

^a^ All values are adjusted for age, energy intake, energy-adjusted fat intake, body weight, education level, household income, physical activity, alcohol drinking status, and smoking status.
*Q1–4*, quartile 1–4; *LDL*, low-density lipoprotein; *HDL* high-density lipoprotein; *Ref*, reference

**Supplementary Table 7. Hazard ratios and 95% confidence intervals (95% CIs), further adjusted for energy-adjusted fat intake for the incidence of dyslipidemia and its components, based on the amino acid intake quartile** ^a^

|  | **Amino acid intake (g/day)** | | | |  |
| --- | --- | --- | --- | --- | --- |
|  | **Q1** | **Q2** | **Q3** | **Q4** | ***P* for trend** |
| **Men** |  |  |  |  |  |
| **Essential amino acid** |  |  |  |  |  |
| Hypertriglyceridemia | Ref | 1.08 (0.86-1.36) | 0.94 (0.72-1.22) | 0.92 (0.64-1.33) | 0.4890 |
| Hypercholesterolemia | Ref | 0.88 (0.71-1.10) | 0.71 (0.55-0.92) | 0.59 (0.41-0.86) | 0.0033 |
| Hyper-LDL cholesterolemia | Ref | 0.87 (0.67-1.12) | 0.67 (0.50-0.90) | 0.65 (0.43-0.98) | 0.0246 |
| Hypo-HDL cholesterolemia | Ref | 1.00 (0.80-1.26) | 0.90 (0.69-1.16) | 0.72 (0.50-1.04) | 0.0546 |
| Dyslipidemia | Ref | 1.00 (0.87-1.14) | 0.85 (0.72-0.99) | 0.76 (0.61-0.94) | 0.0046 |
| **Nonessential amino acid** |  |  |  |  |  |
| Hypertriglyceridemia | Ref | 1.10 (0.87-1.39) | 0.97 (0.74-1.27) | 0.91 (0.63-1.33) | 0.4639 |
| Hypercholesterolemia | Ref | 0.79 (0.63-0.99) | 0.68 (0.52-0.88) | 0.50 (0.34-0.74) | 0.0005 |
| Hyper-LDL cholesterolemia | Ref | 0.82 (0.64-1.06) | 0.62 (0.46-0.84) | 0.56 (0.36-0.85) | 0.0036 |
| Hypo-HDL cholesterolemia | Ref | 0.95 (0.75-1.20) | 0.98 (0.75-1.27) | 0.80 (0.55-1.16) | 0.2692 |
| Dyslipidemia | Ref | 0.95 (0.83-1.10) | 0.86 (0.73-1.00) | 0.73 (0.58-0.91) | 0.0034 |
|  |  |  |  |  |  |
| **Women** |  |  |  |  |  |
| **Essential amino acid** |  |  |  |  |  |
| Hypertriglyceridemia | Ref | 0.99 (0.83-1.18) | 0.88 (0.71-1.07) | 0.77 (0.58-1.02) | 0.0517 |
| Hypercholesterolemia | Ref | 0.99 (0.89-1.10) | 0.87 (0.77-0.98) | 0.85 (0.72-1.01) | 0.0308 |
| Hyper-LDL cholesterolemia | Ref | 1.00 (0.88-1.13) | 0.90 (0.78-1.04) | 0.90 (0.74-1.10) | 0.2103 |
| Hypo-HDL cholesterolemia | Ref | 0.82 (0.66-1.02) | 0.93 (0.73-1.17) | 0.79 (0.57-1.08) | 0.2340 |
| Dyslipidemia | Ref | 0.96 (0.88-1.05) | 0.88 (0.80-0.97) | 0.84 (0.73-0.96) | 0.0046 |
| **Nonessential amino acid** |  |  |  |  |  |
| Hypertriglyceridemia | Ref | 0.99 (0.83-1.19) | 0.87 (0.70-1.07) | 0.79 (0.59-1.05) | 0.0772 |
| Hypercholesterolemia | Ref | 0.92 (0.83-1.03) | 0.86 (0.76-0.97) | 0.78 (0.66-0.92) | 0.0036 |
| Hyper-LDL cholesterolemia | Ref | 0.93 (0.81-1.05) | 0.91 (0.78-1.05) | 0.84 (0.69-1.04) | 0.1124 |
| Hypo-HDL cholesterolemia | Ref | 0.79 (0.64-0.98) | 0.87 (0.69-1.10) | 0.74 (0.54-1.02) | 0.1168 |
| Dyslipidemia | Ref | 0.91 (0.83-0.99) | 0.86 (0.78-0.95) | 0.78 (0.68-0.89) | 0.0003 |

^a^ All values are adjusted for age, energy intake, energy-adjusted fat intake, body weight, education level, household income, physical activity, alcohol drinking status, and smoking status.
*Q1–4*, quartile 1–4; *LDL*, low-density lipoprotein; *HDL* high-density lipoprotein; *Ref*, reference

**Supplementary Table 8. Hazard ratios and 95% confidence intervals for the incidence of dyslipidemia and its components, based on the quartile of amino acid intake per kilogram body weight per day** ^a^

|  | **Amino acid intake (g/kg/day)** | | | |  |
| --- | --- | --- | --- | --- | --- |
|  | **Q1** | **Q2** | **Q3** | **Q4** | ***P* for trend** |
| **Men** |  |  |  |  |  |
| **Essential amino acid** |  |  |  |  |  |
| Hypertriglyceridemia | Ref | 0.89 (0.72-1.09) | 0.85 (0.69-1.05) | 0.73 (0.59-0.91) | 0.0043 |
| Hypercholesterolemia | Ref | 0.88 (0.71-1.08) | 0.77 (0.62-0.95) | 0.67 (0.54-0.83) | 0.0002 |
| Hyper-LDL cholesterolemia | Ref | 0.86 (0.67-1.09) | 0.83 (0.66-1.05) | 0.72 (0.57-0.92) | 0.0116 |
| Hypo-HDL cholesterolemia | Ref | 0.80 (0.65-0.99) | 0.80 (0.65-0.98) | 0.55 (0.44-0.68) | <0.0001 |
| Dyslipidemia | Ref | 0.87 (0.77-0.99) | 0.82 (0.72-0.93) | 0.67 (0.59-0.77) | <0.0001 |
| **Nonessential amino acid** |  |  |  |  |  |
| Hypertriglyceridemia | Ref | 0.94 (0.76-1.17) | 0.87 (0.71-1.08) | 0.81 (0.65-1.00) | 0.0404 |
| Hypercholesterolemia | Ref | 0.91 (0.74-1.12) | 0.79 (0.64-0.98) | 0.69 (0.55-0.86) | 0.0004 |
| Hyper-LDL cholesterolemia | Ref | 0.84 (0.66-1.07) | 0.85 (0.67-1.07) | 0.73 (0.57-0.93) | 0.0153 |
| Hypo-HDL cholesterolemia | Ref | 0.77 (0.62-0.96) | 0.83 (0.68-1.01) | 0.57 (0.46-0.71) | <0.0001 |
| Dyslipidemia | Ref | 0.89 (0.79-1.01) | 0.84 (0.74-0.96) | 0.71 (0.63-0.81) | <0.0001 |
|  |  |  |  |  |  |
| **Women** |  |  |  |  |  |
| **Essential amino acid** |  |  |  |  |  |
| Hypertriglyceridemia | Ref | 0.89 (0.76-1.05) | 0.68 (0.57-0.80) | 0.60 (0.51-0.71) | <0.0001 |
| Hypercholesterolemia | Ref | 0.94 (0.85-1.04) | 0.88 (0.80-0.97) | 0.75 (0.68-0.83) | <0.0001 |
| Hyper-LDL cholesterolemia | Ref | 0.98 (0.87-1.10) | 0.88 (0.78-0.99) | 0.73 (0.65-0.82) | <0.0001 |
| Hypo-HDL cholesterolemia | Ref | 1.00 (0.83-1.22) | 0.84 (0.69-1.02) | 0.77 (0.63-0.94) | 0.0022 |
| Dyslipidemia | Ref | 0.94 (0.87-1.02) | 0.84 (0.78-0.91) | 0.74 (0.68-0.80) | <0.0001 |
| **Nonessential amino acid** |  |  |  |  |  |
| Hypertriglyceridemia | Ref | 0.83 (0.71-0.97) | 0.70 (0.60-0.83) | 0.58 (0.49-0.68) | <0.0001 |
| Hypercholesterolemia | Ref | 0.93 (0.85-1.03) | 0.89 (0.81-0.98) | 0.74 (0.67-0.82) | <0.0001 |
| Hyper-LDL cholesterolemia | Ref | 0.96 (0.85-1.07) | 0.87 (0.77-0.98) | 0.73 (0.64-0.82) | <0.0001 |
| Hypo-HDL cholesterolemia | Ref | 0.95 (0.78-1.15) | 0.84 (0.69-1.02) | 0.78 (0.64-0.94) | 0.0057 |
| Dyslipidemia | Ref | 0.92 (0.85-1.00) | 0.85 (0.79-0.92) | 0.74 (0.68-0.80) | <0.0001 |

^a^ All values are adjusted for age, energy-adjusted fat intake, education level, household income, physical activity, alcohol drinking status, and smoking status.
*Q1–4*, quartile 1–4; *LDL*, low-density lipoprotein; *HDL* high-density lipoprotein; *Ref*, reference

**Supplementary Table 9. Hazard ratios and 95% confidence intervals for the incidence of dyslipidemia and its components, based on the quartile of protein intake per kilogram body weight per day** ^a^

|  | **Protein intake (g/kg/day)** | | | |  |
| --- | --- | --- | --- | --- | --- |
|  | **Q1** | **Q2** | **Q3** | **Q4** | ***P* for trend** |
| **Men** |  |  |  |  |  |
| **Plant-based protein** |  |  |  |  |  |
| Hypertriglyceridemia | Ref | 0.83 (0.67-1.03) | 0.87 (0.71-1.07) | 0.67 (0.54-0.84) | 0.0007 |
| Hypercholesterolemia | Ref | 0.86 (0.70-1.07) | 0.84 (0.68-1.04) | 0.62 (0.50-0.78) | <0.0001 |
| Hyper-LDL cholesterolemia | Ref | 0.96 (0.76-1.22) | 0.85 (0.67-1.09) | 0.67 (0.52-0.87) | 0.0010 |
| Hypo-HDL cholesterolemia | Ref | 1.00 (0.81-1.24) | 0.90 (0.73-1.11) | 0.58 (0.46-0.72) | <0.0001 |
| Dyslipidemia | Ref | 0.90 (0.80-1.03) | 0.88 (0.77-0.99) | 0.65 (0.57-0.74) | <0.0001 |
| **Animal-based protein** |  |  |  |  |  |
| Hypertriglyceridemia | Ref | 1.00 (0.80-1.25) | 1.10 (0.88-1.38) | 0.83 (0.64-1.07) | 0.0986 |
| Hypercholesterolemia | Ref | 0.93 (0.74-1.16) | 0.93 (0.74-1.17) | 0.74 (0.57-0.96) | 0.0165 |
| Hyper-LDL cholesterolemia | Ref | 0.97 (0.75-1.25) | 0.97 (0.75-1.26) | 0.87 (0.65-1.16) | 0.3067 |
| Hypo-HDL cholesterolemia | Ref | 0.94 (0.76-1.16) | 0.82 (0.65-1.03) | 0.78 (0.61-1.00) | 0.0403 |
| Dyslipidemia | Ref | 0.99 (0.86-1.13) | 0.96 (0.84-1.11) | 0.83 (0.71-0.97) | 0.0077 |
|  |  |  |  |  |  |
| **Women** |  |  |  |  |  |
| **Plant-based protein** |  |  |  |  |  |
| Hypertriglyceridemia | Ref | 0.81 (0.68-0.95) | 0.69 (0.58-0.82) | 0.57 (0.48-0.68) | <0.0001 |
| Hypercholesterolemia | Ref | 0.85 (0.77-0.94) | 0.81 (0.73-0.90) | 0.72 (0.65-0.80) | <0.0001 |
| Hyper-LDL cholesterolemia | Ref | 0.87 (0.77-0.98) | 0.79 (0.70-0.89) | 0.69 (0.61-0.78) | <0.0001 |
| Hypo-HDL cholesterolemia | Ref | 1.03 (0.84-1.26) | 0.83 (0.67-1.02) | 0.73 (0.59-0.90) | 0.0003 |
| Dyslipidemia | Ref | 0.89 (0.82-0.97) | 0.80 (0.74-0.87) | 0.72 (0.66-0.78) | <0.0001 |
| **Animal-based protein** |  |  |  |  |  |
| Hypertriglyceridemia | Ref | 0.88 (0.75-1.04) | 0.79 (0.66-0.94) | 0.68 (0.56-0.83) | <0.0001 |
| Hypercholesterolemia | Ref | 0.98 (0.89-1.09) | 0.96 (0.86-1.07) | 0.87 (0.77-0.97) | 0.0069 |
| Hyper-LDL cholesterolemia | Ref | 1.04 (0.92-1.18) | 0.96 (0.84-1.09) | 0.87 (0.76-1.00) | 0.0104 |
| Hypo-HDL cholesterolemia | Ref | 0.99 (0.82-1.21) | 1.04 (0.85-1.28) | 0.90 (0.72-1.13) | 0.3594 |
| Dyslipidemia | Ref | 0.96 (0.88-1.04) | 0.93 (0.85-1.01) | 0.85 (0.77-0.93) | 0.0002 |

^a^ All values are adjusted for age, energy-adjusted fat intake, education level, household income, physical activity, alcohol drinking status, and smoking status.
*Q1–4*, quartile 1–4; *LDL*, low-density lipoprotein; *HDL* high-density lipoprotein; *Ref*, reference
